# Supplementary material for: Mapping evidence on the factors contributing to long waiting times and interventions to reduce waiting times within primary health care facilities in South Africa: A scoping review
Source: PLoS One. 2024 Aug 21;19(8):e0299253. doi: 10.1371/journal.pone.0299253 (PMC11338458; doi:10.1371/journal.pone.0299253)
Supplement: S2 Appendix — (DOCX) [file pone.0299253.s002.docx]

**S2 Appendix: PubMed Search strategy**

| **Keyword search** | **Date of search** | **Search engine** | **Number of publications retrieved** |
| --- | --- | --- | --- |
| ((((("patients"[MeSH Terms] OR "patients"[All Fields] OR "patient"[All Fields]) AND waiting[All Fields] AND ("time"[MeSH Terms] OR "time"[All Fields])) AND (factors[All Fields] AND causing[All Fields] AND waiting[All Fields] AND ("time"[MeSH Terms] OR "time"[All Fields]))) AND ("interventions"[All Fields] AND reduce[All Fields] AND waiting[All Fields] AND ("time"[MeSH Terms] OR "time"[All Fields]))) AND ("primary health care"[MeSH Terms] OR ("primary"[All Fields] AND "health"[All Fields] AND "care"[All Fields]) OR "primary health care"[All Fields] OR ("primary"[All Fields] AND "healthcare"[All Fields]) OR "primary healthcare"[All Fields])) AND ("south africa"[MeSH Terms] OR ("south"[All Fields] AND "africa"[All Fields]) OR "south africa"[All Fields]) AND ("2010/01/01"[PubDate] : "2022/10/10"[PubDate]) | 2022/10/10 | PubMed | 540 |
