# Supplementary material for: Mapping evidence on the factors contributing to long waiting times and interventions to reduce waiting times within primary health care facilities in South Africa: A scoping review
Source: PLoS One. 2024 Aug 21;19(8):e0299253. doi: 10.1371/journal.pone.0299253 (PMC11338458; doi:10.1371/journal.pone.0299253)
Supplement: Attachment — Submitted filename: Response to Reviewers.docx [file pone.0299253.s003.docx]

**Author’s response to the reviews.**

**Manuscript title: Mapping evidence on the factors contributing to long waiting times and interventions to reduce waiting times within primary health care facilities in South Africa: a scoping review**

**Manuscript PONE-D-23-01980**

We are very grateful for the reviews provided by the editor and the reviewers of this manuscript. The comments were very useful. We have revised the manuscript according to the reviewer’s comments. Below is the point-by-point response to the reviewer’s comments. Please find attached a revised version of the manuscript with tracked changes highlighted in ‘red’.

**Reviewer 1**

| **#** | **Editor’s comment** | **Authors’ responses** | **Page and Line Number/s** |
| --- | --- | --- | --- |
| 1 | I strongly recommend to rewrite the introduction which needs to be build more logically. Now, you go from general information to more specific and back. You also often repeat the same information | Thanks for the recommendation, we have rewritten the introduction. | Page 4, lines 74-110 |
| 2 | Also, I would suggest to use a proofreading service because some parts are not written well (not clear enough, grammar, enumeration instead of separate sentences, etc.) | Thanks for the suggestion, we have proofread our manuscript | Whole manuscript |
| 3 | You should also emphasize the novelty of this study | Thanks, this has been emphasized | Page 5, lines 105-110 |
| 4 | RQ: the general RQ should be: “What is known about…?” and the specific RQ should be: “Which factors contribute to an increase in the waiting time? … | Thanks, this has been corrected | Page 6, lines 135-141 |
| 5 | Arksey & O’Malley: normally there are 6 steps. You did not consult the stakeholders. Why not? In this case it seems very interesting to ask them what they think about these results. | The consultation stage is optional in Arksey and O’Malley’s original framework for conducting scoping reviews. However, in this scoping review, this stage will be omitted as the focus is mapping evidence on the factors contributing to long waiting times and interventions to reduce waiting times within primary health care facilities in South Africa, so there will not be any consultation of stakeholders as the stakeholders will be consulted during the qualitative phase of the research. | Page 6 |
| 6 | Repetition in inclusion section and identifying section! | Thanks, this has been corrected | Page 7-8 |
| 7 | What do you mean with “grey literature”. You should at least describe what your definition is and where you have looked for these papers | Thanks, we have clarified our definition of grey literature. | Page 7, lines 153-157 |
| 8 | Discussion: just a summary of the results above, and no comparison with the existing literature. Was this found elsewhere??? | We have updated our discussion section | Pages 19-21 |
| **Reviewer** | **2** |  |  |
| 1 | **Abstract:** |  |  |
| A | Word count is above 300 words. Please shorten | Thanks, the abstract section has been shortened | Pages 2-3, lines 28-54 |
| B | Objective (This scoping review explored and mapped literature on the factors contributing to long waiting times and interventions to reduce waiting times within PHC facilities in South Africa) is mentioned in Methods. Shift to Introduction. | We have removed the sentence from the methods section |  |
| C | Conclusion: Mentions "Therefore, implementing the use of an electronic database within the PHC facilities for effective patient administrative records will enable patients to successfully secure appointments" - Use of only database will not solve the scheduling problem. Thus, the sentence should be rephrased to "implementing the use of an electronic appointment scheduling system and database". This comment holds true for the main text of the manuscript as well. | Thanks for the observation, we have corrected the sentence as suggested | Pages 2-3, line 53 and Page 22, lines 175-179 |
| 2 | Results |  |  |
| a | Page 16, Lines 11-13: "A study reported that communication was another factor that influenced waiting time, hence few of the participants were foreign nationals and did not understand local language/s." - Not clear who the "participant" is here. From the conclusion, it seems the authors wished to write "patients". Rephrase as appropriate. | Thanks, we have rephrased as appropriate | Page 16, lines 39-41 |
| 3 | Conclusions |  |  |
| A | Page 20, Lines 5-6: "The included studies showed that most patients were not satisfied with the amount of time spent in healthcare facilities while waiting for medical assistance." This is new information in Discussion, no corresponding results presented to arrive at this conclusion. | Thanks, the sentence has been corrected | Page 19, lines 112-113 |
| B | Page 20, Lines 23-24: "As a result, some patients would prefer to miss their appointments and go to other clinics where the staff attitude is more favourable and this in turn creates a disorganised patient flow and increased waiting times (40, 42)." his is new information in Discussion, no corresponding results presented to arrive at this conclusion | Thanks, the sentence has been corrected | Page 16, lines 46-47 and Page 20, lines 128-130 |
| C | Page 21, Lines 3-4: "Staff reporting late for work, having unauthorized meal-breaks, preoccupied with non-work related matters and leaving work ahead of time (39, 41)." This is new information in Discussion, no corresponding results presented to arrive at this conclusion. | Thanks, the sentence has been corrected | Page 16, lines 37-39, page 20, lines 131-133 |
|  | Overall, the authors are suggested to include these new statements pertaining to the effects of long waiting times in the results section (and not discussion section for the first time) wherever appropriate. | Thanks, we have included the statements in the result section |  |
